# Virtual reality as an adjunct to anesthesia in the operating room

**DOI:** 10.1186/s13063-019-3922-2

**Published:** 2019-12-27

**Authors:** Adeel Faruki, Thy Nguyen, Samantha Proeschel, Nadav Levy, Jessica Yu, Victoria Ip, Ariel Mueller, Valerie Banner-Goodspeed, Brian O’Gara

**Affiliations:** Department of Anesthesia, Critical Care and Pain Medicine, Beth Israel Deaconess Medical Center, Harvard Medical School, 330 Brookline Ave, Boston, MA 02215 USA

**Keywords:** Propofol, Virtual reality, Monitored anesthesia care, Regional, Upper extremity surgery

## Abstract

**Background:**

Advancements in virtual reality (VR) technology have resulted in its expansion into health care. Preliminary studies have found VR to be effective as an adjunct to anesthesia to reduce pain and anxiety for patients during upper gastrointestinal endoscopies, dental procedures and joint arthroplasties. Current standard care practice for upper extremity surgery includes a combination of regional anesthesia and intraoperative propofol sedation. Commonly, patients receive deep propofol sedation during these cases, leading to potentially avoidable risks of over-sedation, hypotension, upper airway obstruction, and apnea. The objective of this study is to evaluate the effectiveness of VR technology to promote relaxation for patients undergoing upper extremity surgery, thereby reducing intraoperative anesthetic requirements and improving the perioperative patient experience.

**Methods:**

In this single-center, randomized controlled trial, 40 adult patients undergoing upper extremity orthopedic surgery will be randomly allocated to either intraoperative VR immersion or usual care. VR immersion is designed to provide patients with a relaxing virtual environment to alleviate intraoperative anxiety. All patients receive a peripheral nerve block prior to surgery. Patients in the intervention group will select videos or immersive environments which will be played in the VR headset during surgery. An anesthesia provider will perform their usual clinical responsibilities intraoperatively and can administer anesthetic medications if and when clinically necessary. Patients in the control arm will undergo perioperative anesthesia according to standard care practice. The primary outcome is the difference in intraoperative propofol dose between the groups. Secondary outcomes include postoperative analgesia requirements and pain scores, length of stay in the postanesthesia care unit, overall patient satisfaction and postoperative functional outcomes.

**Discussion:**

It is unknown whether the use of VR during upper extremity surgery can reduce intraoperative anesthetic requirements, reduce perioperative complications, or improve the postoperative patient experience. A positive result from this clinical trial would add to the growing body of evidence that demonstrates the effectiveness of VR as an adjunct to anesthesia in reducing intraoperative pain and anxiety for multiple types of procedure. This could lead to a change in practice, with the introduction of a nonpharmacologic intervention potentially reducing the burden of over-sedation while still providing a satisfactory perioperative experience.

**Trial registration:**

ClinicalTrials.gov, NCT03614325. Registered on 9 July 2018.

## Background

Virtual reality (VR) offers novel solutions in health care and has been gradually implemented into medical education, rehabilitation, and management of mental health and chronic pain [1]. VR is believed to create an immersive experience that can restrict the mind from processing acute pain and has been shown to be superior to simpler distraction methods in reducing pain scores for inpatients with any source of pain greater than 3/10 [2]. In preliminary studies, VR has been found to be safe and effective as an adjunct to standard sedative and analgesic protocols to reduce pain and anxiety for patients during upper gastrointestinal endoscopies, dental procedures, dressing changes for burns, and during the first stage of labor [3–6]. To date, it is unknown whether the use of VR during upper extremity surgery as an adjunct to standard anesthetic practice can reduce intraoperative anesthetic requirements and improve the perioperative patient experience.

Currently, the standard anesthesia practice for upper extremity surgical cases involves the combination of regional anesthesia and monitored anesthesia care. The goal of the regional anesthetic is to prevent both sensation and motor function to the operative region, which minimizes pain and discomfort for the patient and improves operating conditions for the surgeon. With an insensate and immobile extremity, patients undergoing upper extremity surgeries ideally would only require additional anesthetic agents to reduce intraoperative anxiety. In daily practice, however, it is common for patients to receive doses of intravenous sedatives such as propofol that may be out of proportion to their requirements for anxiolysis, potentially increasing the risk of over-sedation, hypotension, upper airway obstruction, apnea and postoperative delirium [7–9]. Such avoidable risks are common with excessive sedation and can result in serious morbidity. In fact, 21% of monitored anesthesia care claims in the Closed Claims Database were related to respiratory depression due to an absolute or relative overdose of sedative medications [10]. Using reduced doses of propofol for sedation during hip surgery has been shown to reduce the incidence of postoperative delirium by 50% [9]. Therefore, an intervention that could reduce intraoperative sedative requirements could prove to be valuable in reducing the risk of postoperative adverse events after upper extremity surgery.

The objective of this study is to evaluate the effectiveness of VR technology to reduce intraoperative sedative requirements during upper extremity surgery.

## Methods/design

### Study design

**Fig. 1:**
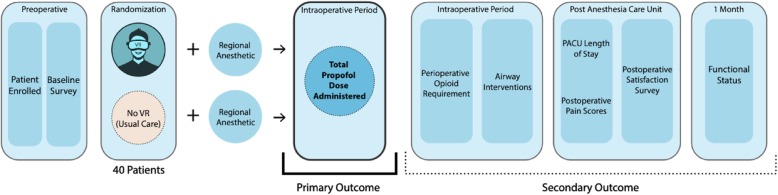
Study schematic. PACU postanesthesia care unit, VR virtual reality

The Virtual Reality in the Operating Room trial is a randomized, controlled, single-center clinical trial of adult orthopedic surgical patients undergoing upper extremity surgery. Individuals will be randomized in a 1:1 allocation to either undergo immersion relaxation via the intraoperative use of VR or usual care. The primary outcome of this study will be the difference in intraoperative propofol dose between the groups. Secondary outcomes include postoperative analgesia dose requirements, postoperative pain scores, length of stay in the postanesthesia care unit (PACU) and overall patient satisfaction. Additionally, at 1 month postoperatively, functional outcomes will be evaluated via telephone administration of the Disabilities of the Arm, Shoulder, and Hand (DASH) assessment. A study schematic is provided in Fig. 1.

### Setting

This study is being conducted at the Beth Israel Deaconess Medical Center (BIDMC) in Boston, Massachusetts, USA. The BIDMC is a 700-bed tertiary care academic facility which serves as a teaching hospital for Harvard Medical School. More than 1800 upper extremity orthopedic surgeries are performed at the BIDMC every year.

### Study registration

**Fig. 2:**
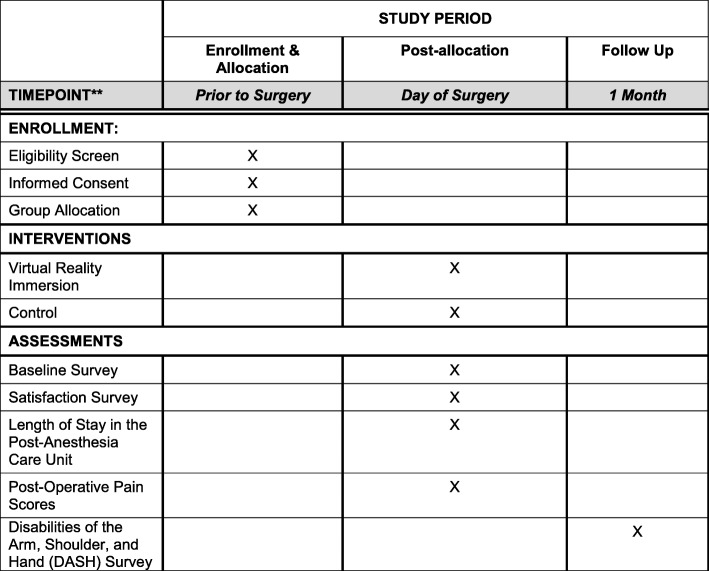
SPIRIT flow diagram

Institutional Review Board (IRB) approval was obtained from the Committee on Clinical Investigations at the BIDMC (IRB protocol number 2018-P-000398). This trial was registered on the National Institutes of Health ClinicalTrials.gov website on 3 August 2018 with the identifier NCT03614325. Upon completion of the trial, results will be reported following the Consolidated Standards of Reporting Trials guidelines and the Standard Protocols Items: Recommendations for Interventional Trials (SPIRIT) checklist (Additional file 1  and Fig. 2) [11, 12]. The trial is currently active and ongoing, and any amendments made to the protocol will be reported to and approved by the BIDMC IRB.

### Inclusion and exclusion criteria

Patients are deemed eligible for trial inclusion if they are due to undergo upper extremity surgery at the BIDMC and receive preoperative regional anesthesia according to standard practice. We will exclude patients who: 1) are under 18 years of age; 2) have an active infection or open wounds of the face or eye area; 3) have a history of seizures of other symptoms linked to an epileptic condition; 4) plan to wear hearing aids during the surgical procedure; 5) have a pacemaker or other implanted medical device; 6) have droplet or airborne precautions; 7) have English language limitations; 8) require deep sedation; or 9) are deemed ineligible to the approach by the surgeon. Patients who do not speak English will be excluded, as clear verbal communication will be essential during the study procedures. Patients will be approached preoperatively in the surgery clinic or via telephone. Patients will not be offered compensation for participation in the trial.

### Randomization

Following informed consent, patients are randomized in a 1:1 allocation using block randomization of equal sizes to either intraoperative VR immersion or usual care control. The Research Electronic Data Capture (REDCap) randomization module will be used to implement the study randomization scheme prior to any study interventions taking place. REDCap is a secure, web-based application that supports data capture for research studies and allows customization to support the development of the electronic case report form [13]. Each patient is randomized via REDCap software after enrollment. The data manager will input the patient into REDCap after enrollment and determine into which group the patient will be enrolled. The randomization sequence is unknown to research members ahead of time. The allocation will only be informed to research members after the patient is enrolled and assigned. Considering the nature of the intervention, treatment allocation is unblinded; therefore, a protocol for unblinding participants is not applicable.

### Drop-out criteria

After providing informed consent, the patient can voluntarily withdraw from the study. Additionally, if the anesthesia team or investigators determine that the patient is no longer eligible to participate as a result of cancelled surgery or meeting an exclusion criterion, the patient will be withdrawn from the study.

### Study intervention: virtual reality immersive relaxation

Patients randomized to the VR immersion group will be asked to wear the VR headset and view the programming of their choice for the entire procedure as an adjunct to standard anesthesia practice. The software used in this study was created by VR Health USA, and is designed to promote relaxation and calmness. Some of the immersive VR environments that have been created with the software include sitting on a beach, in a peaceful meadow or forest, or on a mountaintop. In addition, a library of short videos is available for patients to view via a web-based user interface. A study team member will be available at all times intraoperatively to assist with the device programming and technical issues. An anesthesia provider will perform their usual clinical responsibilities, including closely monitoring the patient throughout the procedure, and is free to administer anesthetic medications if clinically necessary or when requested by the patient. Both the patient and the anesthesia provider can decline to continue with the VR programming at any time. This is not considered a drop-out criterion and the patient’s results will be analyzed based on the intention-to-treat. At the conclusion of the surgery, the VR headset will be removed and postoperative care will begin according to the current standard care practice.

### Control arm: usual care

Patients randomized to the control group will undergo perioperative anesthesia according to the current standard care practice at the BIDMC.

### Clinical management in both groups

In both groups, patients will receive regional anesthesia preoperatively in accordance with standard practice. Patients may receive anxiolytic medications such as midazolam during the placement of the nerve block at the discretion of the regional anesthesia team. All intraoperative anesthetic management and medication administration decisions will be determined by the treating provider. All patients will be monitored (vital signs and capnography) according to American Society of Anesthesiologist standards.

Multiple data points, including airway interventions and use of airway assist devices, will be abstracted from the anesthesia medical record.

### Data collection

To assess primary and secondary outcomes, data on intraoperative propofol dose, length of PACU stay, administration of postoperative analgesia, pain scores and patient satisfaction will be collected. Additionally, information regarding patient demographics (including, but not limited to, age, gender, race, body mass index and comorbidities), surgical characteristics, intraoperative vital signs, medication administration and postoperative outcome data will be reviewed from patients’ medical records. Postoperative pain scores, documented on a scale of 0 to 10 by nursing staff in the PACU, will be abstracted from the medical records. There are no plans to prevent drop-outs or loss to follow-up during the trial. All study data will be stored anonymously and managed in a REDCap database hosted at the BIDMC. The database is audited and monitored for timeliness, completeness and accuracy.

Research team members met on a regular basis to discuss enrollment of patients. The data manager/statistician (AM) and data quality manager met regularly to assess and monitor the data quality. With a trial of this size and the low risk to participants, the study design team members did not feel it was necessary to have a safety monitoring or steering committee. No interim analysis or trial audit will be performed during the trial. The trial is low risk for harm and therefore no post-trial care or compensation provisions are needed for trial participants experiencing harm from their participation.

Missing data from the perioperative course is not anticipated as the patients will undergo anesthesia with an anesthetic and medical record. If the patient is lost to follow-up 1 month postprocedure then their data will not include DASH assessment information.

### Reporting of compliance and adverse events

Since this study utilizes a noninvasive device, we expect the associated risks to be minimal. According to the device manufacturers, 1 in 4000 people experiencing VR may have severe dizziness, seizures, eye or muscle twitching, or blackouts triggered by light flashes. These symptoms are more common in children and young people [14]. To mitigate these risks, we are excluding patients under 18 years of age and patients with a history of seizures from this study. This patient population is undergoing surgery and it is expected that they may have a number of unrelated adverse health events during their hospital course. Therefore, we will limit the scope of adverse event monitoring and reporting to those believed to be related to the study procedures. Participants will be monitored for protocol compliance and occurrence of adverse events until the time of PACU discharge. If a participant experiences any symptoms of severe dizziness, seizures, eye or muscle twitching, or blackouts triggered by light flashes, the VR programming will be stopped and the patient will be treated accordingly. These symptoms will be recorded as adverse events.

### Statistical analysis

#### Sample size calculation

Based on preliminary data from a related study evaluating the use of VR immersion for major joint surgery, we anticipate that we may be able to achieve a 30% reduction in intraoperative propofol dose between groups [15]. Using a two-sided α of 0.05, 80% power, an effect size of 30%, with mean propofol doses of 155 mg/h (standard deviation (SD) 45 mg/h) and 108.5 mg/h (SD 45 mg/h) we anticipate needing a sample size of 32 patients. To account for anticipated drop-out, we will add 8 patients to the calculated sample size for a total planned enrollment of 40 patients, with 20 in each of the two groups.

#### Data analysis

We will use SAS software version 9.3 or later (SAS Institute, Cary, North Carolina, USA) to conduct all analyses. Descriptive statistics of the data will be evaluated and presented using mean (± SD) and median (interquartile range) for variables not normally distributed. Continuous data will be compared using parametric or nonparametric *t* tests as appropriate. Categorical data will be expressed using proportions and compared using a Chi-square test or Fisher’s Exact test.

#### Analysis of primary outcome

Our primary outcome, total intraoperative propofol dose, will be normalized to the duration of the procedure. It will be analyzed as a continuous variable and differences in dose between groups will be assessed using a *t* test or Wilcoxon Rank Sum test, depending on the normality of the data. Although randomization is expected to account for potential differences between groups, we will use univariate and multivariable linear regression modeling to adjust for differences that persist after randomization, if necessary. The analysis of the data will not be performed until all data have been collected, at which point the primary outcome of total intraoperative propofol dose, the secondary outcomes of PACU length of stay, medications given in PACU, pain scores in PACU, satisfaction surveys and DASH assessment will all have been documented into the anesthesia and medical records or into REDCap. Therefore, the statistical analysis cannot be biased by the data analyst and thus it is not necessary to blind the data analyst.

#### Analysis of secondary outcomes

Secondary outcomes including length of PACU stay, doses of medications administered, and pain scores will be expressed as continuous variables. Secondary outcomes with continuous variables will be assessed using a *t* test or Wilcoxon Rank Sum test to compare the two groups, depending on the normality of the data.

Doses of medications will be documented during PACU length of stay as total milligrams dosed. Medications include dexmedetomidine, propofol, remifentanil, hydromorphone, fentanyl, midazolam, morphine, intravenous acetaminophen, ondansetron and ketorolac. The total dose of each medication given will be analyzed as a continuous variable.

Patient satisfaction will be evaluated from a REDCap survey using quantitative scales to assess the level of agreement with prompted statements regarding pain, nausea, anxiety and overall experience, using slide bars with visible anchors of ‘strongly disagree’ (0), ‘neutral’ (50) and ‘strongly agree’ (100) (Additional file 4). The data from the survey will be used as continuous variables for analysis.

We will evaluate adherence to the randomization assignment and, should any patient receive an intervention different from their randomization assignment, a per-protocol analysis will be performed as an additional secondary analysis. Therefore, the study team will analyze the patient for the intervention received instead of the randomization assignment. Functional success of the surgery will be assessed using the DASH questionnaire (Additional file 4) at 1 month postsurgery and differences between groups will be analyzed [16].

## Discussion

This study will be the first to evaluate the potential for VR technology to reduce intraoperative propofol requirements for patients undergoing upper extremity surgery. Patients undergoing upper extremity surgery are at risk for over-sedation, potentially resulting in postoperative adverse events. By decreasing the amount of propofol sedation patients receive, the risk of adverse events may be reduced. A positive result of this study would add to the growing body of evidence that demonstrates the effectiveness of VR as an adjunct to standard sedative and analgesic protocols in reducing pain and anxiety. Possible uses for VR intraoperatively include patients with significant anxiety and patients who are at increased risk for complications during deep sedation secondary to medical comorbidities such as severe pulmonary hypertension.

Our study has several limitations. First, for this proof-of-concept trial we will not blind anesthesiologists to patient assignment. We acknowledge that this may potentially introduce bias to our primary outcome as it is possible the anesthesiologist in the operating room may administer a different dose of propofol solely based on whether the patient is wearing the VR headset and not based on the required dose for patient comfort during surgery. Another limitation of this study is selecting intraoperative propofol dose as the primary outcome. Although a significant reduction in dose would be statistically relevant, this may not directly correlate with clinically relevant outcomes (e.g., reduced complication rates). Despite these limitations, we chose total intraoperative propofol dose as our primary outcome because it is an objective, quantifiable measure for which we performed a robust power calculation. Since propofol has the ability to cause dose-dependent respiratory depression, we believe it would be clinically plausible that a sizeable reduction in propofol dose could decrease the risk of airway complications. For this proof-of-concept trial, it would be impractical to recruit the large number of patients needed at a single center to accurately detect a significant difference in perioperative complication rates. Therefore, we thought it best to first perform a randomized controlled trial powered to assess a significant reduction in propofol dosing after the implementation of VR. The results of this trial could be used to aid in the design of a larger clinical trial aimed at investigating whether the use of intraoperative VR can influence clinical outcomes.

There are various potential limitations with VR technology that may contribute to a nonimmersive environment, such as device connectivity issues. This could possibly interfere with patient relaxation and subsequent satisfaction if commonly encountered. Another potential limitation is that patients are unable to interact directly with the software. Our study is also susceptible to selection bias since patients who enroll voluntarily understand they potentially will not receive the standard care of intraoperative propofol anesthetic. As such, the cohort of patients enrolling into the study may have less anxiety than the general population at baseline, influencing the generalizability of our secondary outcomes such as patient satisfaction. Lastly, as with any study at a single institution, there is a possibility that our results may not be generalizable.

To date, it is unknown whether the use of VR during upper extremity surgery can reduce intraoperative anesthetic requirements, reduce perioperative complications, or improve the perioperative patient experience. A successful result of the proposed trial could spur future investigations which could lead to a change in practice, with the introduction of a nonpharmacologic, patient-led intervention to potentially reduce the burden of over-sedation while still providing a satisfactory perioperative experience.

## Supplementary information

**Additional file 1.** SPIRIT checklist.

**Additional file 2.** WHO Trial Registration Data Set - Structured Summary.

**Additional file 3.** Informed Consent Form.

**Additional file 4.** Surveys and DASH Questionnaire.

## Data Availability

Data from the current study is available from the corresponding author upon reasonable request. The study team will have access to and will maintain the final trial dataset.

## Abbreviations

BIDMC: Beth Israel Deaconess Medical Center
DASH: Disabilities of the Arm, Shoulder and Hand
IRB: Institutional Review Board
PACU: Postanesthesia care unit
REDCap: Research Electronic Data Capture
SD: Standard deviation
SPIRIT: Standard Protocols Items: Recommendations for Interventional Trials
VR: Virtual reality
