# Supplementary material for: Virtual reality as an adjunct to anesthesia in the operating room
Source: Trials. 2019 Dec 27;20:782. doi: 10.1186/s13063-019-3922-2 (PMC6935058; doi:10.1186/s13063-019-3922-2)
Supplement: Supplementary file 2 — Additional file 2. WHO Trial Registration Data Set - Structured Summary. [file 13063_2019_3922_MOESM2_ESM.pdf]

Virtual Reality in the Operating Room: Using Immersive Relaxation as an Adjunct to Anesthesia

Thank you for your participation in our study. We would like to ask you a few questions about your experience during the study.

Please indicate which group you were assigned to

- ◯ Virtual Reality
- ◯ Control Group

Please rate your agreement with the following:

I felt that the VR headset was comfortable.	Strongly Disagree	Neutral	Strongly Agree
		(Place a mark on the scale above)	
I enjoyed the selection of VR programs/environments.	Strongly Disagree	Neutral	Strongly Agree
		(Place a mark on the scale above)	
The VR program was easy to use.	Strongly Disagree	Neutral	Strongly Agree
		(Place a mark on the scale above)	
My pain level was controlled during surgery.	Strongly Disagree	Neutral	Strongly Agree
		(Place a mark on the scale above)	
I felt relaxed during my procedure.	Strongly Disagree	Neutral	Strongly Agree
		(Place a mark on the scale above)	
I felt anxious during my procedure.	Strongly Disagree	Neutral	Strongly Agree
		(Place a mark on the scale above)	
I felt nauseated during my procedure.	Strongly Disagree	Neutral	Strongly Agree
		(Place a mark on the scale above)	
I remember being aware of how I felt while I was in the operating room for my procedure.	Strongly Disagree	Neutral	Strongly Agree
		(Place a mark on the scale above)	
I would be interested in a VR program if I was going to have another surgery.	Strongly Disagree	Neutral	Strongly Agree
		(Place a mark on the scale above)	

I think that VR should be used for patients undergoing hand or wrist surgery.	◯ True ◯ False ◯ Unsure
Do you have any suggestions for environments or programs you might be interested in?	_____
Are there any aspects of this study that you would change for future participants?	☐ Increase the number of relaxation modules<br>☐ Change the type of VR headset used<br>☐ Other
If other, please describe what you might change	_____
Please rate your overall satisfaction with this study.	Strongly Disagree Neutral Strongly Agree<br>*(Place a mark on the scale above)*
Please indicate any other feedback you have for the research team	_____

Patient Satisfaction Survey - Version Date: 03/20/2019

For Study Staff:

VRHealth Study ID: _____

Date and Time of Survey Administration: _____ (MM/DD/YYYY) at _____:_____

Person providing responses (circle one):
Patient
Surrogate: _____ (relation to patient)

Person recording responses: _____

Additional notes (if applicable):
