# Supplementary material for: Virtual reality as an adjunct to anesthesia in the operating room
Source: Trials. 2019 Dec 27;20:782. doi: 10.1186/s13063-019-3922-2 (PMC6935058; doi:10.1186/s13063-019-3922-2)
Supplement: Supplementary file 3 — Additional file 3. Informed Consent Form. [file 13063_2019_3922_MOESM3_ESM.pdf]

Virtual Reality in the Operating Room: Using Immersive Relaxation as an Adjunct to Anesthesia

Baseline Survey

Thank you for your participation in our study. We would like to ask you a few questions.

Have you ever used a VR headset before?	◯ Never<br>◯ Once<br>◯ A few times<br>◯ Many times
Have you ever been interested in using a VR headset?	◯ Yes<br>◯ No<br>◯ I am unsure.
I think that VR should be used for patients undergoing hand or wrist surgery.	◯ True<br>◯ False<br>◯ Unsure

Please respond to each item about your surgery by marking one box per row. In the past 7 days....

	Never	Rarely	Sometimes	Often	Always
I felt fearful.	◯	◯	◯	◯	◯
I found it hard to focus on anything other than my anxiety.	◯	◯	◯	◯	◯
My worries overwhelmed me.	◯	◯	◯	◯	◯
I felt uneasy.	◯	◯	◯	◯	◯
I felt nervous.	◯	◯	◯	◯	◯
I felt like I needed help for my anxiety.	◯	◯	◯	◯	◯
I felt anxious.	◯	◯	◯	◯	◯
I felt tense.	◯	◯	◯	◯	◯

Baseline Survey - Version Date: 03/20/2019

For Study Staff:

VRHealth Study ID: __________

Date and Time of Survey Administration: _________ (MM/DD/YYYY) at _____:_____

Person providing responses (circle one):
Patient
Surrogate: _______________ (relation to patient)

Person recording responses: ________________________

Additional notes (if applicable):
