# Supplementary material for: Virtual reality as an adjunct to anesthesia in the operating room
Source: Trials. 2019 Dec 27;20:782. doi: 10.1186/s13063-019-3922-2 (PMC6935058; doi:10.1186/s13063-019-3922-2)
Supplement: Supplementary file 4 — Additional file 4. Surveys and DASH Questionnaire. [file 13063_2019_3922_MOESM4_ESM.pdf]

DASH - Disabilities of the Arm, Shoulder and Hand

Study ID

____
(ex. H999)

This is to be completed 1 month after surgery.

Date of DASH Survey Administration ______________________

Name of Staff Recording Responses ______________________

Additional Notes (if applicable) ______________________

INSTRUCTIONS

This questionnaire asks about your symptoms as well as your ability to perform certain activities.

Please answer every question, based on your condition in the last week, by circling the appropriate number.

If you did not have the opportunity to perform an activity in the past week, please make your best estimate on which response would be the most accurate.

It doesn't matter which hand or arm you use to perform the activity; please answer based on your ability regardless of how you perform the task.

Please rate your ability to do the following activities in the last week by clicking the button below the appropriate response.

	1 - NO DIFFICULTY	2 - MILD DIFFICULTY	3 - MODERATE DIFFICULTY	4 - SEVERE DIFFICULTY	5 - UNABLE
1. Open a tight or new jar.	○	○	○	○	○
2. Write.	○	○	○	○	○
3. Turn a key.	○	○	○	○	○
4. Prepare a meal.	○	○	○	○	○
5. Push open a heavy door.	○	○	○	○	○
6. Place an object on a shelf above your head.	○	○	○	○	○
7. Do heavy household chores (e.g., wash walls, wash floors).	○	○	○	○	○
8. Garden or do yard work.	○	○	○	○	○
9. Make a bed.	○	○	○	○	○
10. Carry a shopping bag or briefcase.	○	○	○	○	○

11. Carry a heavy object (over 10 lbs).	○	○	○	○	○
12. Change a lightbulb overhead.	○	○	○	○	○
13. Wash or blow dry your hair.	○	○	○	○	○
14. Wash your back.	○	○	○	○	○
15. Put on a pullover sweater.	○	○	○	○	○
16. Use a knife to cut food.	○	○	○	○	○
17. Recreational activities which require little effort (e.g., cardplaying, knitting, etc.).	○	○	○	○	○
18. Recreational activities in which you take some force or impact through your arm, shoulder or hand (e.g., golf, hammering, tennis, etc.).	○	○	○	○	○
19. Recreational activities in which you move your arm freely (e.g., playing frisbee, badminton, etc.).	○	○	○	○	○
20. Manage transportation needs (getting from one place to another).	○	○	○	○	○
21. Sexual activities.	○	○	○	○	○

	1 - NOT AT ALL	2 - SLIGHTLY	3 - MODERATELY	4 - QUITE A BIT	5 - EXTREMELY
22. During the past week, to what extent has your arm, shoulder or hand problem interfered with your normal social activities with family, friends, neighbours or groups? (click a button)	○	○	○	○	○

	1 - NOT LIMITED AT ALL	2 - SLIGHTLY LIMITED	3 - MODERATELY LIMITED	4 - VERY LIMITED	5 - UNABLE
23. During the past week, were you limited in your work or other regular daily activities as a result of your arm, shoulder or hand problem? (click a button)	○	○	○	○	○

Please rate the severity of the following symptoms in the last week. (click a button)

	1 - NONE	2 - MILD	3 - MODERATE	4 - SEVERE	5 - EXTREME
24. Arm, shoulder or hand pain.	○	○	○	○	○
25. Arm, shoulder or hand pain when you performed any specific activity.	○	○	○	○	○
26. Tingling (pins and needles) in your arm, shoulder or hand.	○	○	○	○	○
27. Weakness in your arm, shoulder or hand.	○	○	○	○	○
28. Stiffness in your arm, shoulder or hand.	○	○	○	○	○

	1 - NO DIFFICULTY	2 - MILD DIFFICULTY	3 - MODERATE DIFFICULTY	4 - SEVERE DIFFICULTY	5 - SO MUCH DIFFICULTY THAT I CAN'T SLEEP
29. During the past week, how much difficulty have you had sleeping because of the pain in your arm, shoulder or hand? (click a button)	○	○	○	○	○

	1 - STRONGLY DISAGREE	2 - DISAGREE	3 - NEITHER AGREE NOR DISAGREE	4 - AGREE	5 - STRONGLY AGREE
30. I feel less capable, less confident or less useful because of my arm, shoulder or hand problem.(click a button)	○	○	○	○	○

Calculated DASH Score ________________________________

DASH DISABILITY/SYMPTOM SCORE = [(sum of n responses)/n - 1] x 25, where n is equal to the number of completed responses.

A DASH score may not be calculated if there are greater than 3 missing items.

© INSTITUTE FOR WORK & HEALTH 2006. ALL RIGHTS RESERVED.
